# Simultaneous targeting of 5-LOX-COX and EGFR blocks progression of pancreatic ductal adenocarcinoma

**DOI:** 10.18632/oncotarget.5396

**Published:** 2015-09-28

**Authors:** Chinthalapally V. Rao, Naveena B. Janakiram, Venkateshwar Madka, Vishal Devarkonda, Misty Brewer, Laura Biddick, Stan Lightfoot, Vernon E. Steele, Altaf Mohammed

**Affiliations:** ^1^ Center for Cancer Prevention and Drug Development, Department of Medicine, Hem-Onc Section, Stephenson Cancer Center, University of Oklahoma Health Sciences Center, Oklahoma City, OK, USA; ^2^ Division of Cancer Prevention, Chemopreventive Agent Development Research Group, National Cancer Institute, Bethesda, MD, USA

**Keywords:** chemoprevention, inflammation, EGFR, pancreatic cancer

## Abstract

Cyclooxygenase-2 (COX-2), 5-Lipoxygenase (5-LOX), and epidermal growth factor receptor (EGRF) are over-expressed in human pancreatic ductal adenocarcinoma (PDAC). Using next-generation sequencing (NGS) analysis, we show significant increase in COX-2, 5-LOX, and EGFR expression during PDAC progression. Targeting complementary pathways will achieve better treatment efficacy than a single agent high-dose strategy that could increase risk of side effects and tumor resistance. To target COX-2, 5-LOX, and EGFR simultaneously, we tested effects of licofelone (dual 5-LOX-COX inhibitor), and gefitinib (EGFR inhibitor), individually and in combination, on pancreatic intraepithelial neoplasms (PanINs) and their progression to PDAC using genetically engineered mice. Individually, licofelone (L) and gefitinib (G) significantly inhibited incidence of PDAC in male (72% L, 90% G, *p* < 0.0001) and female (90% L, 85% G, *p* < 0.0001) mice. The combination drug treatment produced complete inhibition of PDAC in both genders. Pancreata of mice receiving combination treatment showed significantly fewer Dclk1-positive cancer stem-like cells, inhibition of COX-2, 5-LOX, PCNA, EGFR and β-catenin expression (*p* < 0.05–0.0002), increased p21 expression. Significant changes in tumor immune responses and desmoplastic reaction was observed by NGS analysis in combination treatment (*p* < 0.05). In summary, early simultaneous targeting of 5-LOX-COX- and EGFR pathways may provide additive inhibitory effects leading to complete suppression of PDAC.

## INTRODUCTION

Pancreatic cancer is usually diagnosed at late stages, with almost uniform lethality. The five-year survival rate for this deadly cancer is <7% [1]. Although gemcitabine is the drug of choice, survival following treatment is marginally improved, by only a few weeks. Hence, new chemopreventive and therapeutic treatment strategies are urgently needed. Research toward understanding this disease led to the identification of major risk factors and important molecular changes underlying pancreatic cancer initiation and progression.

The initiation of pancreatic cancer lesions and their progression to pancreatic ductal adenocarcinoma (PDAC) and further metastatic invasion are associated with inflammation. Several lines of evidence show that cyclooxygenase-2 (COX-2) and 5-lipoxygenase (5-LOX) are significantly over-expressed in cancers [2–5]. COX-2 and 5-LOX metabolites play a pivotal role in cell signaling and proliferation [2–5]. Their release has been demonstrated in response to epidermal growth factor (EGF) and growth stimuli. Preclinical studies show that COX-2 inhibitors may inhibit pancreatic cancer *in vitro* and *in vivo*; however, those studies have not been translated for clinical use [2–4]. Moreover, use of celecoxib, a COX-2 inhibitor, in the prevention of adenomatous polyps was associated with a significant increase in risk of cardiovascular (CV) events [6, 7]. In another study, the relative risk of CV events was 1.30 with the use of celecoxib, compared with placebo [8]. It is widely accepted that selectively blocking COX-2 will shift arachidonic acid metabolism towards the 5-LOX pathway, overproducing leukotrienes and leading to increased prothrombotic effects [2, 9]. Thus, preclinical and clinical data underscore the importance of arachidonic acid metabolism-related inflammatory responses in pancreatic tumorigenesis.

**Figure 1:**
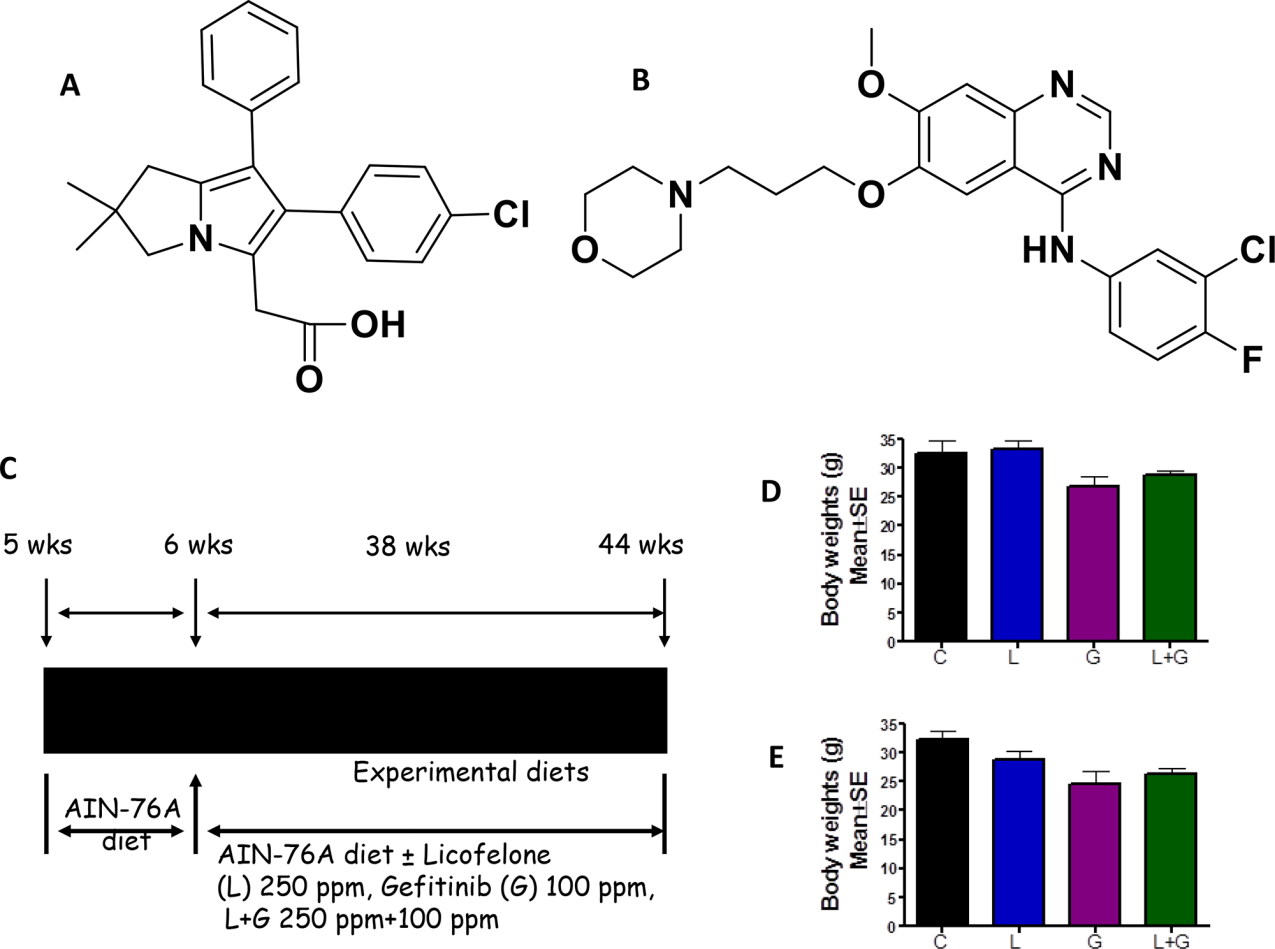
Experimental design for evaluation of licofelone and gefitinib efficacy in PC prevention in GEM **A–B.** Structure of licofelone (A) and gefitinib (B) **C.** Experimental design for evaluation of licofelone, gefitinib, and a combination of licofelone (L) + gefitinib (G) efficacy in PC inhibition in male and female p48^Cre/+^-LSL-Kras^G12D/+^ GEM mice. At six weeks of age, groups of mice (24–34/group for activated p48^Cre/+^-LSL-Kras^G12D/+^ or 12/group for wild-type) were continuously fed AIN-76A diets containing 0, 250 ppm licofelone, or 100 ppm gefitinib, alone or in combination, for 38 weeks. Each pancreas was evaluated histopathologically for marker expression as described in the text. D-E. Effect of licofelone, gefitinib, or the combination on bodyweight (BW; means ± *SE*) at termination of the experiment in male **D.** and female **E.** mice. No statistically significant differences were observed between control and drug-treated p48^Cre/+^-LSL-Kras^G12D/+^ or wild-type mice.

Both COX-2 and EGFR are known to synergistically activate oncogenic signaling. COX-2 and epidermal growth factor receptor (EGFR) were found to be significantly expressed in pancreatic tumors. The expression of COX-2 and EGFR in the majority of undifferentiated pancreatic carcinomas suggests that COX-2 and EGFR might represent targets for adjuvant therapy in anaplastic pancreatic cancer [10]. The activation of the EGFR pathway promotes transcription of the COX-2 gene. Also, EGFR transcription and phosphorylation are activated by the COX-2 pathway. Since both these pathways are involved in inflammation and EGFR signaling that will lead to tumor cell growth, use of agents that combined inhibit both pathways simultaneously may provide synergy for improved tumor inhibition. Although EGFR inhibitors are in use, their benefits are limited and the use of higher doses leads to undesirable side effects and toxicities. Clinically, targeting inflammation by selective COX-2 inhibitors leads to increased cardiovascular and GI toxicities. Therefore, targeting both EGFR and 5-LOX-COX- simultaneously may be an effective approach to modulate three pathways and their downstream signaling, which may result in an increased treatment response. In the present study, we evaluated the potential anti-tumor effects of licofelone, a novel dual 5-LOX-COX- inhibitor (Fig. 1A), and gefitinib, a known EGFR inhibitor (Fig. 1B), either individually or in combination, by simultaneously modulating multiple targets (COX, 5-LOX, EGFR) using a genetically engineered Kras mouse model (GEM) of pancreatic cancer. This model has histopathology and molecular features similar to human pancreatic cancers [11]. The use of this mouse model has played a key role in the elucidation of the mechanisms and underlying pancreatic tumorigenesis, and in the identification of anti-tumor agents for the prevention of pancreatic cancer [12–18].

## RESULTS

### General observations

All GEM and wild-type mice were fed modified AIN-76A diets, with or without licofelone, gefitinib, or a combination (Fig. 1C). There were no significant differences in the bodyweights of control and licofelone-treated animals (Fig. 1D, 1E). However, a non-significant decrease was observed in the bodyweights of animals treated with gefitinib and the combination (Fig. 1D, 1E). Examination of the gross anatomy of GEM and wild-type mice revealed no visible evidence of any abnormality of the kidneys, liver, spleen, intestines, heart, or lungs. There were no significant differences in the weights of these organs in control and experimental-diet-fed mice, indicating that the agents did not produce any overt toxicity. However, pancreata from control-diet-fed GEM developed visible tumors and weighed significantly more than those from the control-diet-fed wild-type mice, which were completely free from tumor growth. Histopathological analysis of formalin-fixed tumors confirmed the presence of PanIN lesions and ductal adenocarcinoma in the pancreata of GEM. Thus, we observed organ-specific tumor growth due to specific p48^Cre/+^ activation of Kras^G12D/+^ in pancreata of GEM, which could be monitored for the potential effects of the test agents administered in diet.

### Inhibition of pancreatic tumor weight and PDAC incidence

**Figure 2:**
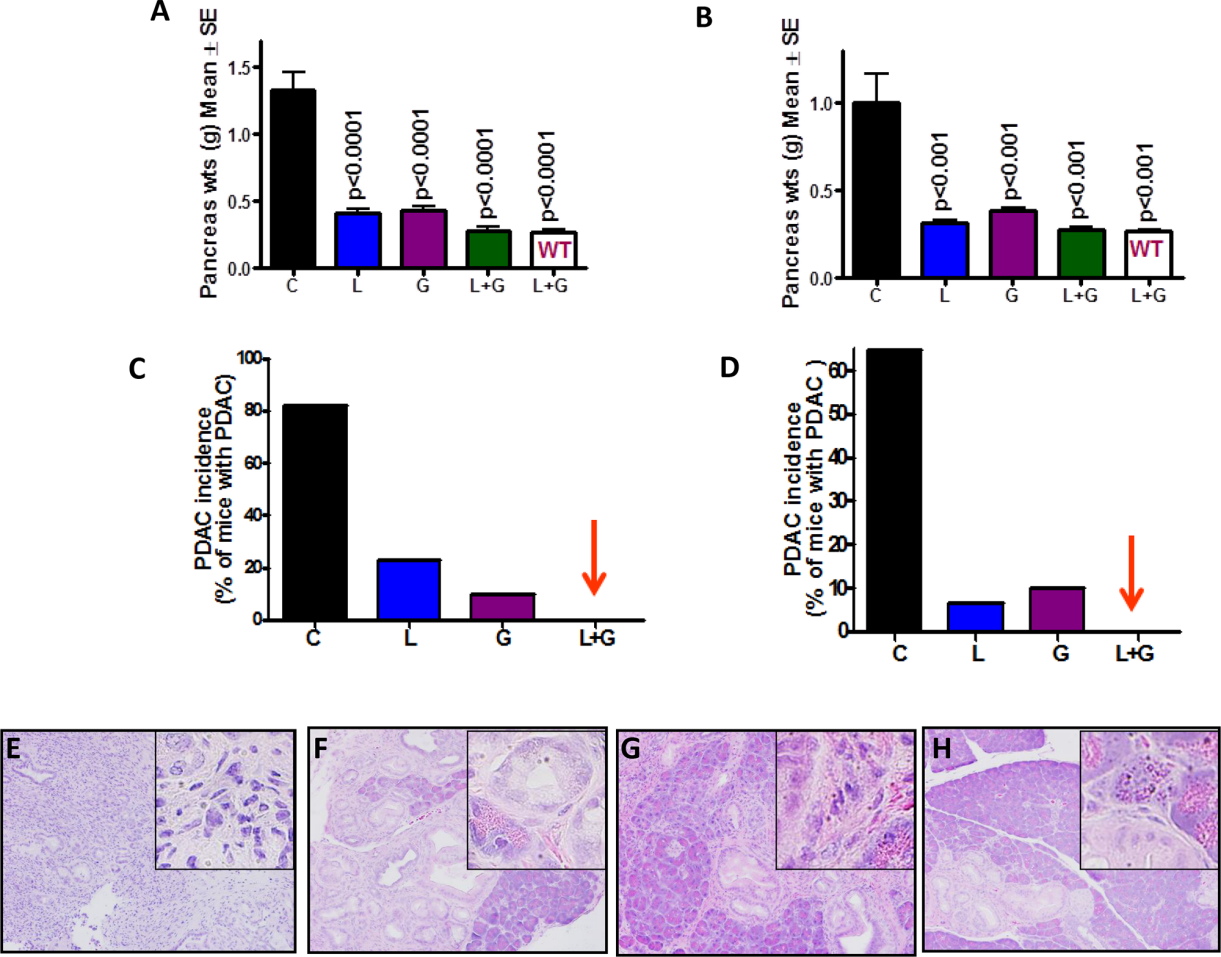
Effect of L, G and L+G on pancreatic tumor weights and PDAC incidence Effect of L, G, and L+G on pancreas weight at the termination of the experiment in male **A.** and female **B.** mice. L+G significantly reduced pancreatic tumor weights. C-D. Effect of L, G, and L+G on the incidence of PDAC in male **C.** and female **D.** mice. L+G showed complete inhibition of PDAC. E-H. Histopathologic analysis of untreated and treated pancreata using Hematoxylin & Eosin staining. Pancreata from untreated animals showing poorly differentiated adenocarcinoma with some of the cells invading stroma **E.** Pancreata from animals treated with L **F.** G **G.** and L+G **H.** showing PanIN lesions.

Pancreatic weight is a simple marker to assess tumor progression [17, 18]. A significant increase in pancreas weight was observed in p48^Cre/+^-LSL-Kras^G12D/+^ mice compared with wild-type mice (Fig. 2A). As summarized in Figs. 2A and 2B, the combination treatment caused a significant decrease in the weight of pancreatic tumors in p48^Cre/+^-LSL-Kras^G12D/+^ mice. The pancreatic tumor weights in untreated control male and female GEM were (Mean ± *SEM*) 1.33 ± 0.13 and 1.005 ± 0.16 g, respectively. In GEM fed licofelone (L), gefitinib (G), or L+G, the pancreas weights were Mean ± *SEM* 0.40 ± 0.03, 0.43 ± 0.27, and 0.27 ± 0.03 g, respectively, in males, and 0.31 ± 0.08 g, 0.38 ± 0.02 g, and 0.27 ± 0.18 g, respectively, in females. The pancreata of wild-type mice treated with L+G weighed 0.26 ± 0.02 g in male mice and 0.26 ± 0.01 g in female mice. The pancreatic tumor weights were reduced by 80% (*p* < 0.0001) with L+G treatment in male mice and by 72% (*p* < 0.001) in female mice (Fig. 2A, 2B). Extensive histopathologic analysis of the pancreas using H&E-stained slides revealed no microscopic pathologic alterations in wild-type mice fed either AIN-76A or L+G-supplemented diets. In contrast, AIN76A-diet-fed male and female p48^Cre/+^LSL-Kras^G12D/+^ mice demonstrated 82% and 64% incidence of PDAC, respectively (Fig. 2C and 2D). L, G, and L+G treatments decreased PDAC incidence to 23%, 10%, and 0%, respectively, in male GEM, and to 6%, 10%, and 0%, respectively, in female GEM (Fig. 2C and 2D). The combination treatment of L+G completely inhibited carcinoma incidence in both male and female GEM. Fig. 2E–2H shows the H&E staining of the pancreatic tumors with and without licofelone, gefitinib, and the combination treatment.

### Combination of licofelone and gefitinib inhibits PanIN lesion progression and carcinoma

**Figure 3:**
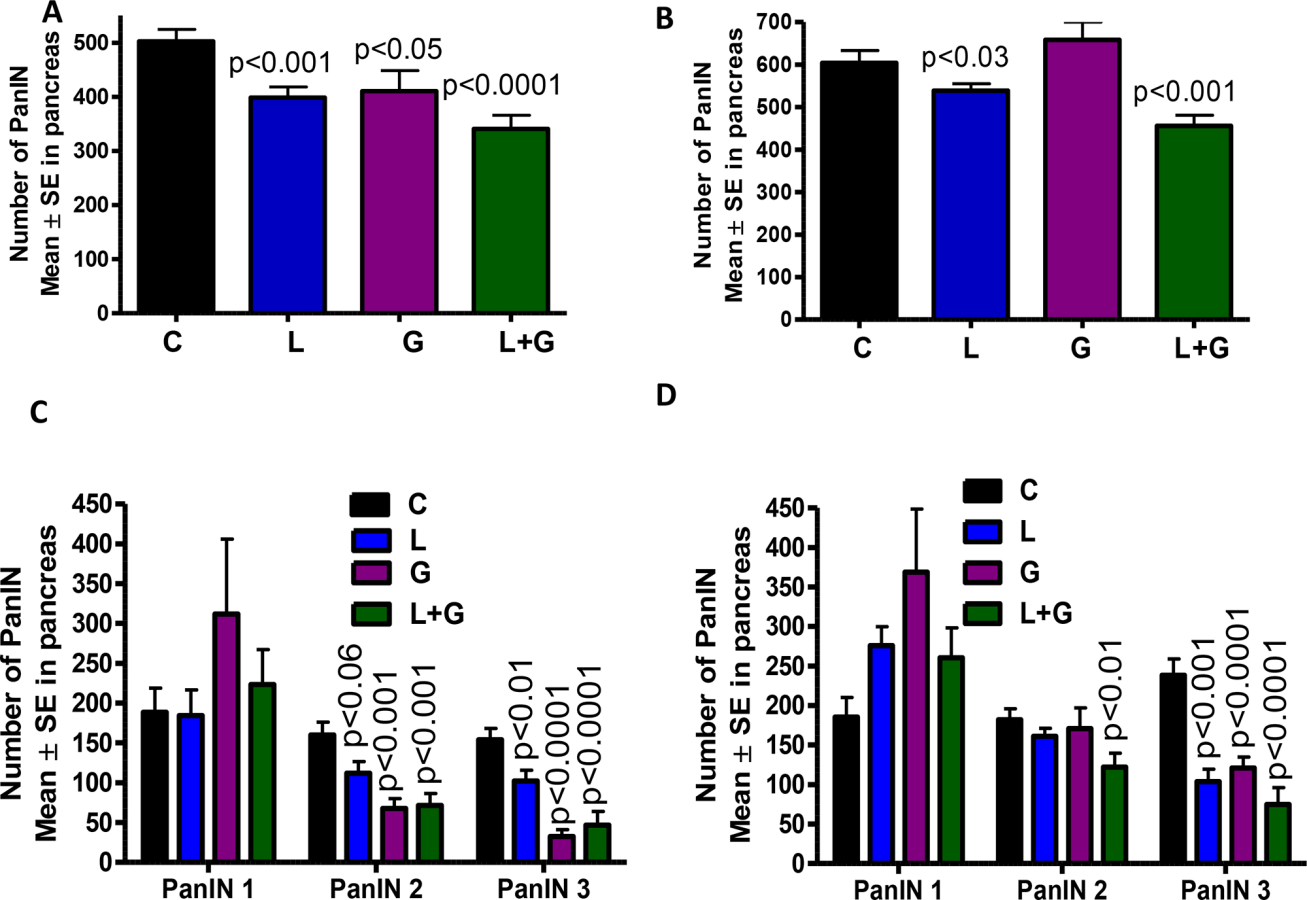
A–D. Effect of L, G, and L+G on PanIN multiplicity in male (A,C) and female (B,D) GEM mice (means ± *SE*)

The total numbers of PanIN lesions in GEM fed AIN-76A diet were (means ± *SE*) 502 ± 22 in male GEM and 604 ± 29 in female GEM. In the treatment groups, total PanINs in male mice were 398 ± 19 (licofelone), 410 ± 38 (gefitinib), and 340 ± 25 (combination), while total PanINs in female mice were 539 ± 16 (licofelone), 658 ± 42 (gefitinib), and 456 ± 25 (combination; Fig. 3A, 3B). In male GEM fed AIN-76A diet, PanINs by lesion classification were (means ± *SE*): 188 ± 30 (PanIN 1), 160 ± 16 (PanIN 2), and 154 ± 16 (PanIN 3). In licofelone-treated male mice, PanIN numbers were 184 ± 32 (PanIN 1), 112 ± 14 (PanIN 2), and 102 ± 13 (PanIN 3). In male mice treated with gefitinib, PanIN numbers were 310 ± 94 (PanIN 1), 67 ± 12 (PanIN 2), and 32 ± 8 (PanIN 3). Finally, male mice receiving the combination treatment had the following PanINs: 223 ± 43 (PanIN 1), 71 ± 15 (PanIN 2), and 46 ± 17 (PanIN 3; Fig. 3C).

The number of PanIN lesions in female GEM fed AIN-76A diet were (means ± SE): 185 ± 24 (PanIN 1), 181 ± 14 (PanIN 2), and 238 ± 20 (PanIN 3). In licofelone-treated female mice, PanIN numbers were 275 ± 23 (PanIN 1), 161 ± 10 (PanIN 2), and 103 ± 15 (PanIN 3). In female mice treated with gefitinib, Pan IN numbers were 368 ± 79 (PanIN 1), 170 ± 26 (PanIN 2), and 120 ± 14 (PanIN 3). Finally, female mice receiving the combination treatment had the following PanIN results: 260 ± 37 (PanIN 1), 122 ± 17 (PanIN 2), and 74 ± 21 (PanIN 3; Fig. 3D).

**Figure 4:**
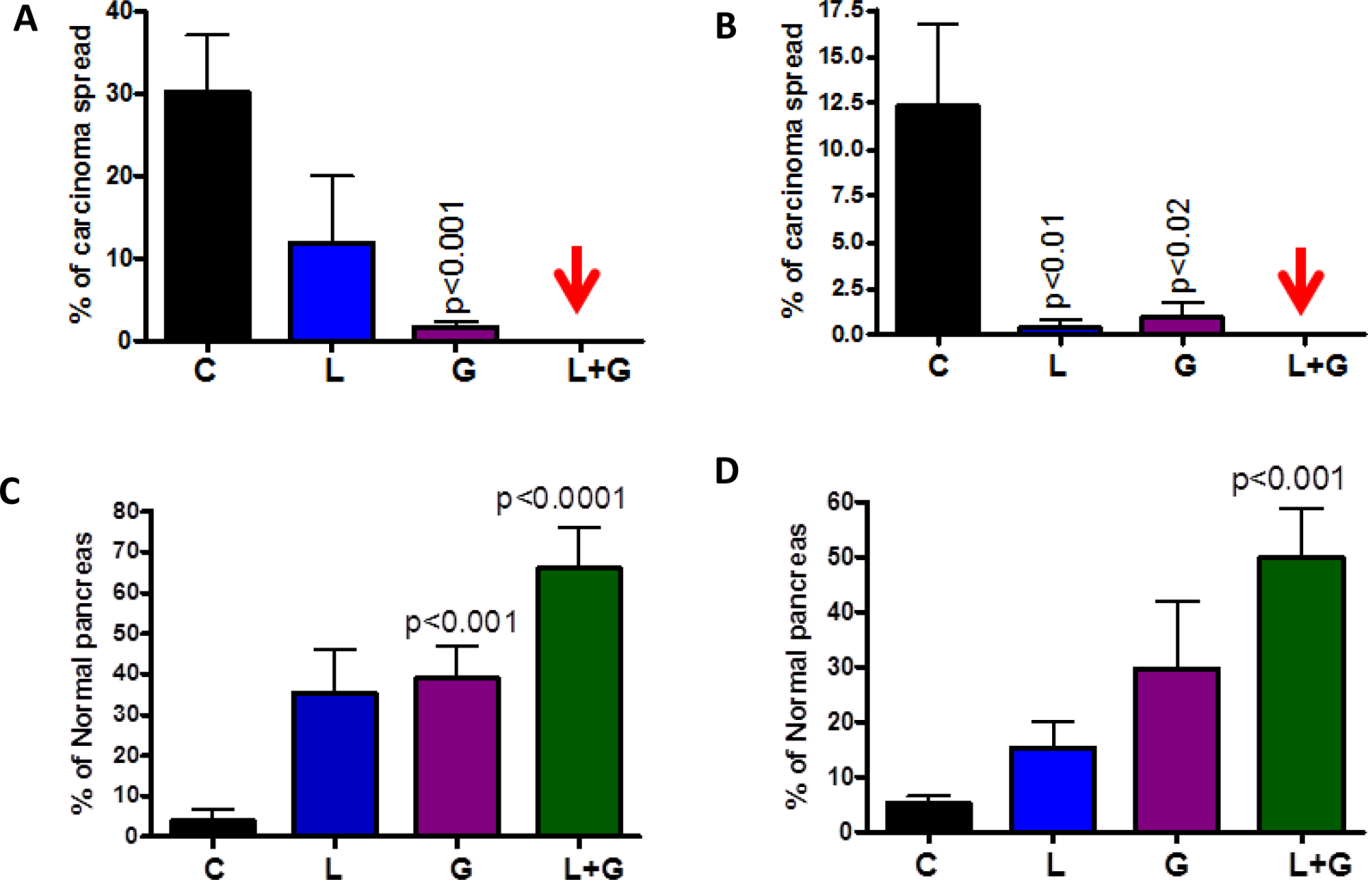
Effect of L, G and L+G on carcinoma percentage **A–B.** Effect of L, G, and L+G on the percentage of pancreata with carcinoma (A-male, B-female). **C–D.** Effect of L, G, and L+G on the percentage of normal appearing pancreata (C-male, D-female). The data in the panels were analyzed by unpaired *t*-test with Welch's correction. Values are considered statistically significant at *p* < 0.05.

The number of PanIN 2 and PanIN 3 lesions or carcinoma *in situ* was suppressed by ~70% in the combination-treated groups (Fig. 3C, 3D). A slight increase in the number of PanIN 1 lesions was observed in pancreata of mice treated with the combination, suggesting a potential blockade of further progression of these lesions to carcinoma *in situ* and PDAC. Carcinoma within the pancreas was completely inhibited in male and female GEM (Fig. 4A, 4B) receiving the combination treatment. Up to 70% of the pancreata from mice treated with the combination of licofelone and gefitinib appeared normal, i.e., free from PanIN lesions and carcinoma, whereas up to 4–5% of pancreata appeared normal in untreated GEM (Fig. 4C, 4D).

### Combination of licofelone and gefitinib inhibits tumor cell proliferation, induces apoptosis, and modulates inflammatory and EGFR signaling

**Figure 5:**
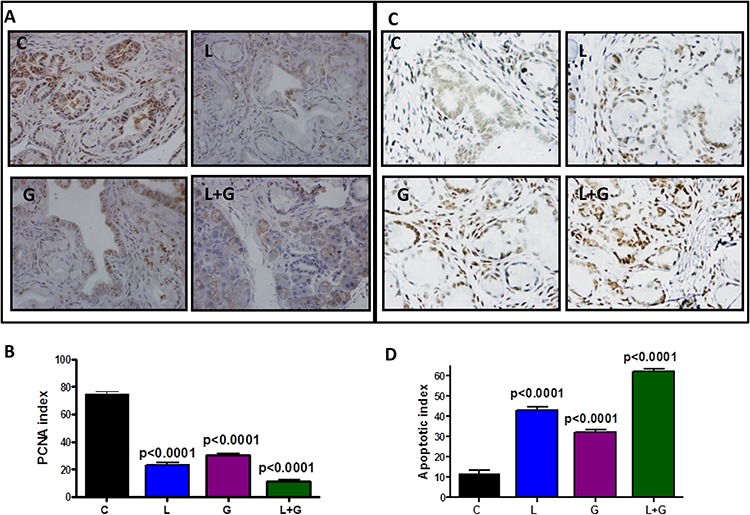
Effect of L, G, and L+G on tumor cell proliferation and apoptosis **A.** Immunohistochemical staining for PCNA in pancreatic tumors from GEM fed control diet or treated with L, G, or L+G. **B.** A significant difference was observed in the proliferative index between combination-treated and control group pancreata. **C–D.** TUNEL assay was done for apoptotic cells in pancreatic tumors from GEM fed control diet or treated with L, G, or L+G (*n* = 6 mice/group). A significant induction of apoptosis was observed in tumors from treated mice compared with tumors from untreated mice.

**Figure 6:**
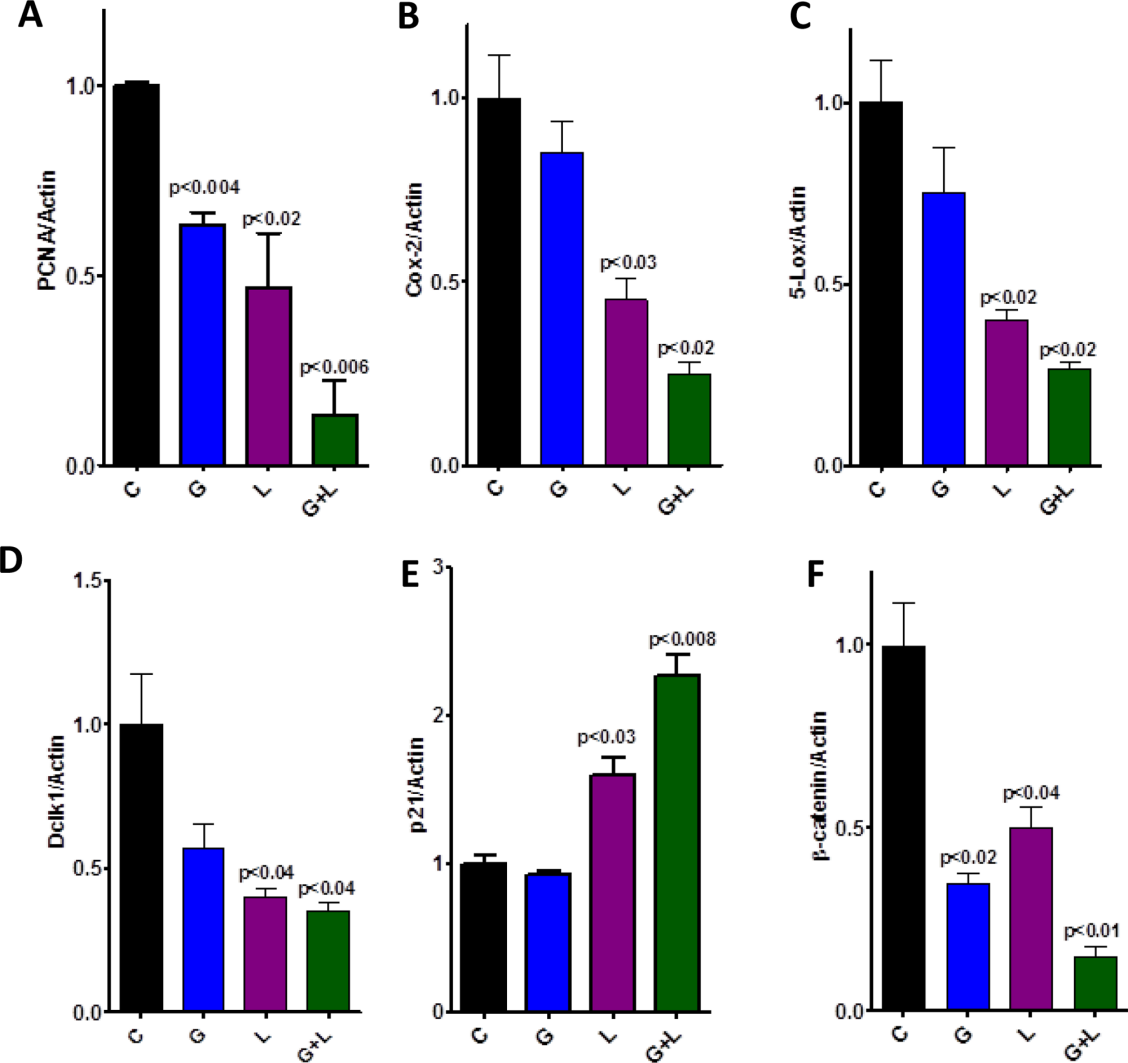
Effect of L, G, and L+G on protein expression of PCNA, COX-2, 5-LOX, DclK1, p21, and β-catenin, as determined by western immunoblotting The combination of L+G significantly decreased PCNA, COX-2, 5-LOX, DclK1, and β-catenin expression, with an increase in p21 expression, compared with individual drug treatment.

**Figure 7:**
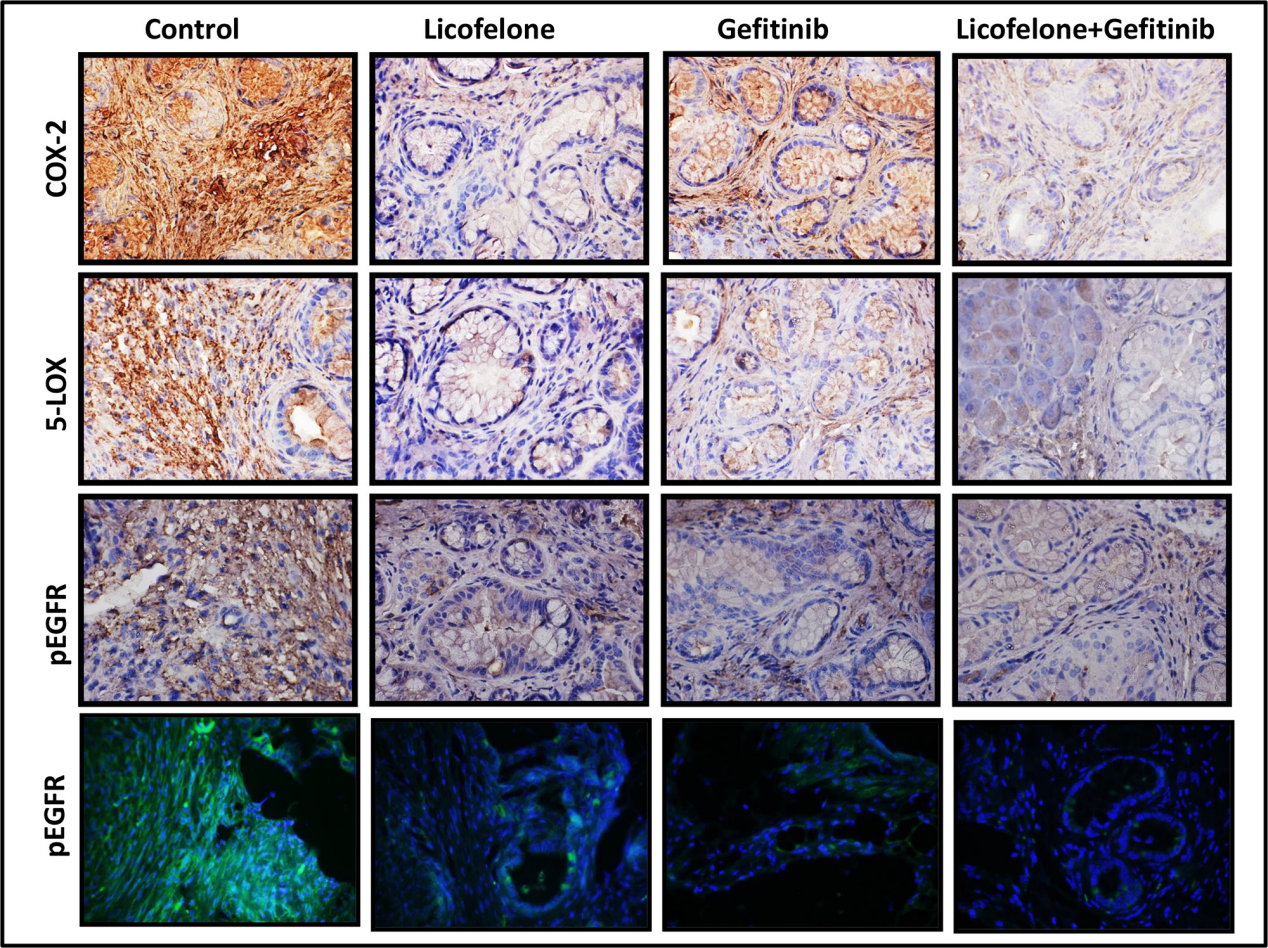
Effect of L, G, and L+G on COX-2, 5-LOX, and p-EGFR Immunohistochemistry (COX-2, 5-LOX, p-EGFR) and immunofluorescence (bottom row) revealed reduced COX-2, 5-LOX, and p-EGFR expression with individual drug treatment and the combination.

**Figure 8:**
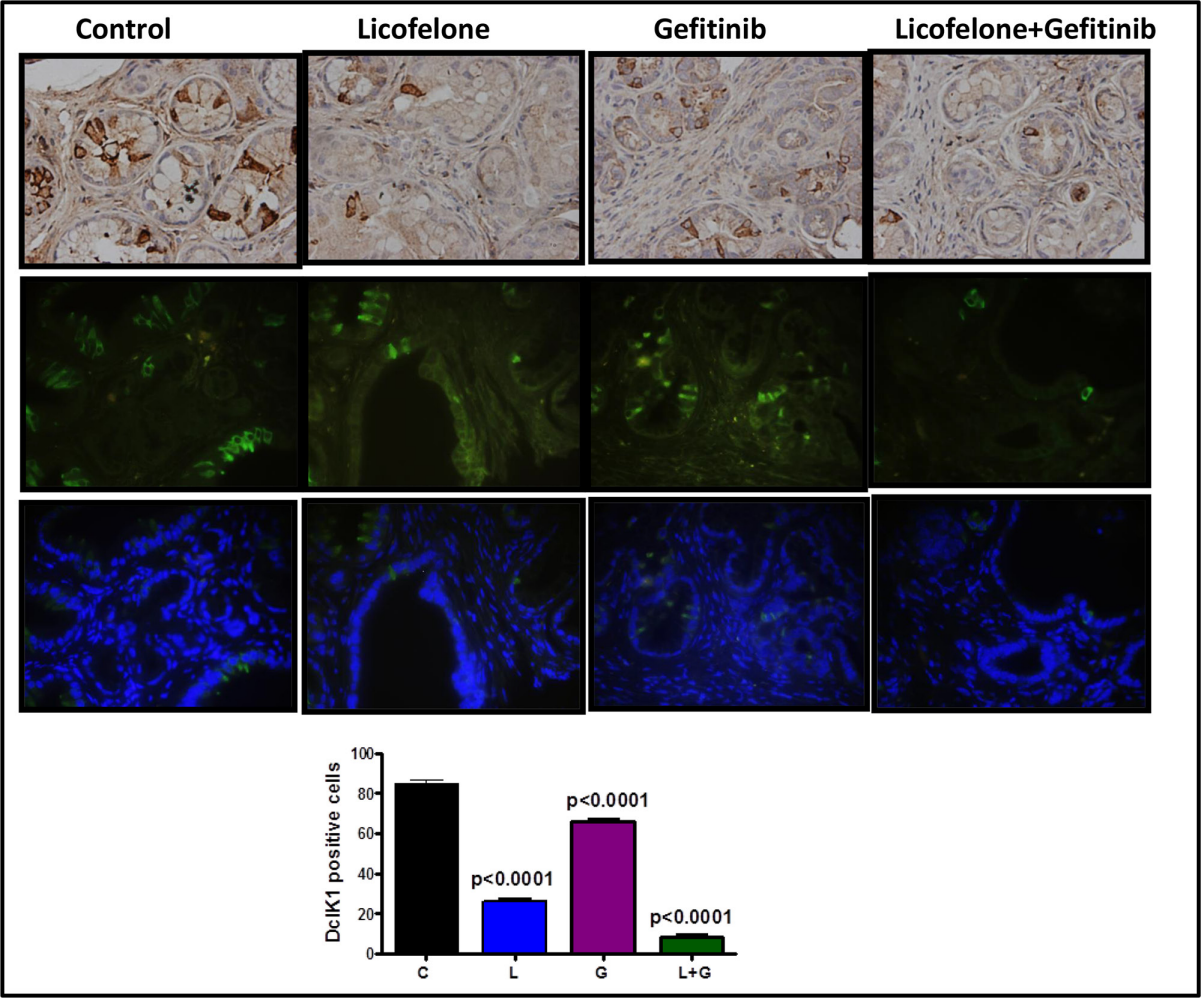
Effect of L, G, and L+G on CSC marker DclK1 Immunohistochemistry (brown-DclK1; top panel) and immunofluorescence (bottom panels; green-DclK1, blue-DAPI) staining for DclK1 in pancreatic tumors from GEM fed control diet or treated with L, G, or L+G. A significant decrease in the number of DclK1-positive cells was observed with the combination treatment compared with individual drug treatment and controls.

IHC/IHF, western immunoblotting, real-time PCR, and terminal deoxynucleotidyl transferase dUTP nick end labeling (TUNEL) assay approaches were used to determine the effects of the drug combination on tumor cell proliferation, apoptosis, and modulation of inflammatory and EGFR signaling in the pancreatic tissues of GEM (Fig. 5–8, Supplementary Fig. 1). IHC staining showed that PanIN lesions/carcinoma were labeled positively for PCNA in p48^Cre/+^-LSL-Kras^G12D/+^ mice fed AIN-76A diet alone (Fig. 5A). Markedly decreased numbers of PCNA-positive cells and proliferative index were observed in mice fed a licofelone+gefitinib combination-supplemented diet compared with individual drug-treated and control-diet-fed mice (Fig. 5A, 5B). Figs. 5C and 5D summarize the effects of licofelone, gefitinib, and the combination on tumor cell apoptosis.

Qualitative microscopic examination showed a substantial increase in TUNEL-positive cells in the pancreatic tumor tissue of mice treated with the combination. Quantification of TUNEL-positive cells from pancreata of control diet–fed mice showed 18 ± 1.8 (mean ± *SEM*) compared with 62 ± 1.4 TUNEL-positive cells in pancreatic tumor tissue from combination-treated mice, accounting for a fivefold increase in the apoptotic index ( *p* < 0.0001, Fig. 5D). Further, we also observed a significant decrease in expression of PCNA, COX-2, 5-LOX, and DclK1 in the combination treatment groups (Fig. 6A–6D, Supplementary Fig. 1). In agreement with the apoptosis results, we observed a significant increase in p21 expression (Fig. 6E, Supplementary Fig. 1) and a decrease in β-catenin in the treatment groups (Fig. 6F, Supplementary Fig. 1). IHC/IHF analysis revealed decreased COX-2, 5-LOX, and pEGFR expression in licofelone+gefitinib treated groups compared with untreated groups (Fig. 7).

### Licofelone + gefitinib combination inhibits DclK1-positive stem-like cells

We have previously shown that DclK1 is a potential stem cell marker for pancreatic carcinogenesis [5]. By inhibiting COX-2, 5-LOX, cytokines, and their receptors, anti-inflammatory agents can block signals from tumor cells, and potentially affect cancer stem cells (CSCs) [2]. We have seen a significant decrease in DclK1 expression in treatment groups (Fig. 8). Combination treatment significantly reduced DclK1 expression (Fig. 8, Supplementary Fig. 1).

### Gene expression profiling in pancreatic tumor tissues

**Figure 9:**
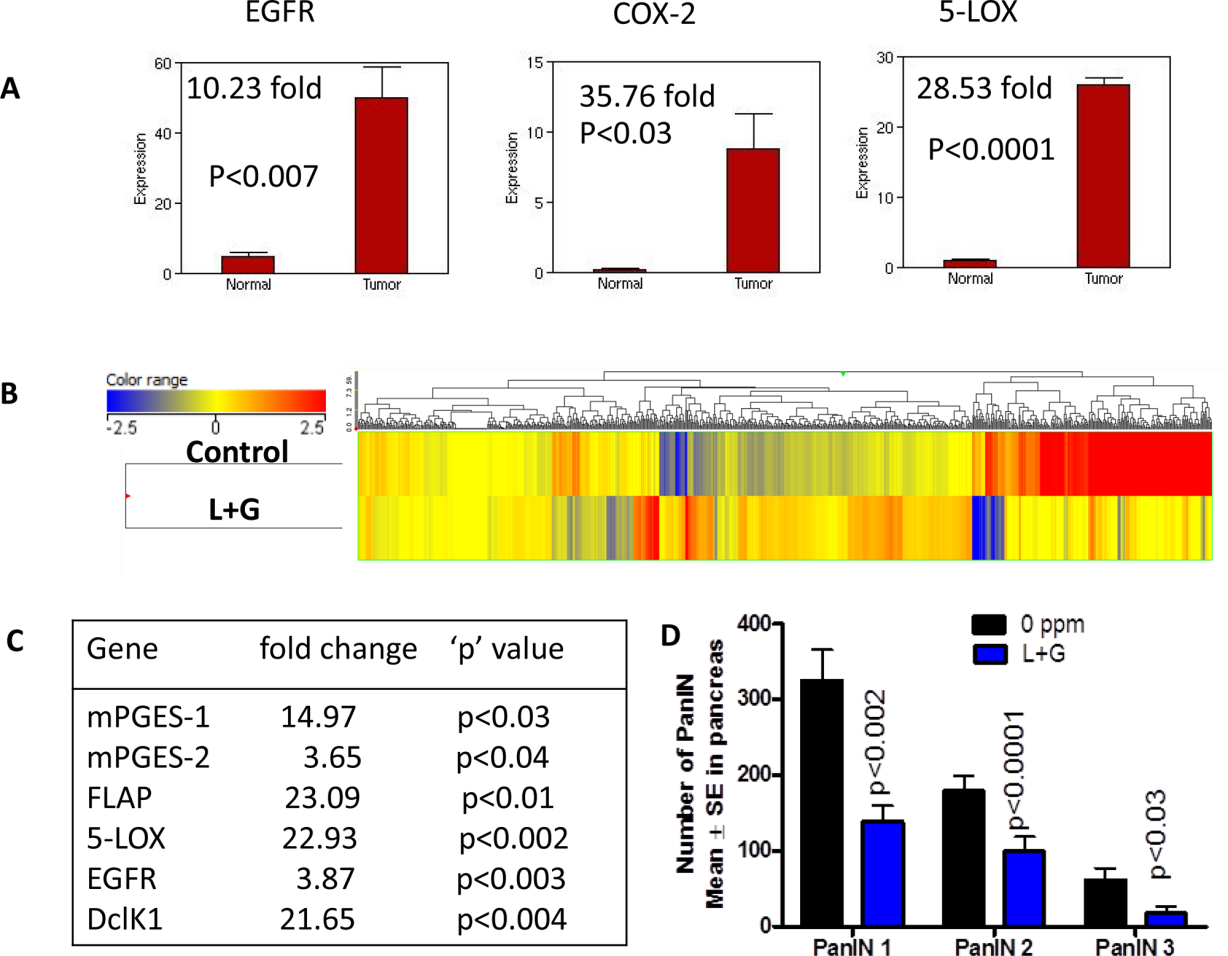
Impact of combination treatment on transcriptomic gene changes **A.** Whole genome transcriptome analysis by Illumina sequencing showing increased mRNA expression of EGFR, COX-2, and 5-LOX in pancreata from GEM mice compared with wild-type mice. **B.** Heat map of the untreated and combination treatment groups showing differential gene expressions. **C.** Transcriptome gene changes in the down regulated mRNA expression of mPGES-1, mPGES-2, FLAP, 5-LOX, EGFR, and DclK1 upon combination treatment. **D.** Combination treatment significantly decreased number of PanIN lesions.

We observed a significant increase in the inflammatory genes COX-2 and 5-LOX, along with EGFR, in the pancreatic tumors from 44-week-old mice compared with pancreata from wild-type mice, as determined by Next Generation Sequencing/RNAseq analysis (Fig. 9A). However, in the individual and combination drug treatments, these levels were significantly reduced (Fig. 6, 7, Supplementary Fig. 1). To investigate transcriptomic changes in actively progressing pancreatic cancer, we performed Next Generation Sequencing/RNAseq using the pancreatic tissues from mice fed control diet or combination drug treatment for 25 weeks. At the endpoint, PanIN lesions and carcinomas were recorded. The sample numbers met the requirements for statistical significance per *t*-test and Benjamini's and Hochberg tests (3–6 per group). Overall, the gene expression profiles showed a pattern of differences between the control and combination treatment groups in the heat map determined by Strand analysis (Fig. 9B). Further, using the GeneSifter Next generation sequencing data set, we identified differentially expressed genes. There were total 7043 hits with a 1.5-fold expression difference threshold, *p* < 0.05 (both upregulated and downregulated genes). The gene data sets 5-LOX activating protein (FLAP), 5-LOX, mPGES-1, mPGES-2, EGFR, and DclK1 were significantly decreased by the combination treatment during the progression of pancreatic lesions to ductal adenocarcinoma (Fig. 9C). Importantly, the combination treatment significantly reduced PanIN1, 2 and 3 lesions (Fig. 9D). These results indicate that both inflammation and EGFR signaling can be tightly regulated by these drugs during PDAC development.

### Impact of combination treatment on tumor immune responses and desmoplastic reaction

Furthermore, the NGS data revealed that dual combination treatment significantly impacted and reduced the matrix metalloproteases (MMP); MMP19, MMP23, MMP9, MMP3, MMP17, MMP12, MMP28 and MMP11 (Table 1). Of all the MMPs, MMP19 was greatly reduced (>24 fold) followed by MMP23 (>21 fold). Notable changes were observed in the genes related to immune responses and desmoplastic reaction (Table 2) along with changes in inflammatory genes (Fig. 9C, Table 2). Importantly, the macrophage stimulating receptor (Mst1r) was reduced by > 40 fold (Table 2). Other genes that were upregulated were galanin recptor 1 (Galr1) (8.96 fold; *p* < 0.05), tumor suppressor glycine N-methyl transferase Gnmt (4.3 fold; *p* < 0.02) and fructosamine 3 kinase (Fn3k) (8.24 fold; *p* < 0.01).

**Table 1:** Transcriptome gene changes in the downregulated mRNA expression of MMPs in response to combination treatment

Gene	fold change	‘*p*’ value
MMP 19	24.32	<0.002
MMP 23	21.48	<0.02
MMP 9	18.47	<0.008
MMP 3	12.04	<0.02
MMP 17	11.47	<0.04
MMP 12	10.39	<0.002
MMP28	10.10	<0.002
MMP 11	7.27	<0.02

**Table 2:** Transcriptome changes in the downregulated mRNA expression of genes related to tumor immune responses and desmoplastic reaction upon combination treatment

Gene	fold change	‘*p*’ value
IL-1β	13.26	<0.002
Mst1r	40.06	<0.02
TGFβr1	6.09	<0.02
TGFβr2	14.09	<0.03
Fibronectin 1	18.85	<0.03
PDGFβ	3.53	<0.02
PDGFrb	6.26	<0.05
VEGFa	1.61	<0.03
CSF-1	5.27	<0.002
SDF-2	3.24	<0.01
CD44	19.82	<0.05
IL-6ra	4.03	<0.02
PD1 (PDCD1)	6.80	<0.02
CD68	9.99	<0.047
CD163	7.26	<0.045

## DISCUSSION

Pancreatic cancer treatment is challenging, due to the aggressive nature of the disease and a lack of effective drug treatments. The commonly used FDA-approved drug, gemcitabine, a deoxycytosine analog, represents the current standard-of-care for advanced PC; gemcitabine improves quality of life in a minority of patients [19] and increases survival by few weeks. Studies showed that median survival is extended by 14 days with the use of EGFR inhibitor erlotinib [20]. Understanding pancreatic cancer development and finding ways to treat this disease at all stages are clear and imperative needs [21].

It is well-established that 95% of the pancreatic cancers are associated with K-ras mutations. Several of the molecular signaling changes occur as the disease progress from initial pancreatic precursor lesions PanINs (PanIN 1 to PanIN 2 to PanIN 3) to ductal adenocarcinoma. Of these, the most commonly observed changes are marked dysregulation of EGFR signaling and inflammatory pathways. EGFR dysregulation is observed in over 87% of patients with pancreatic cancer [22–24]. Both inflammation and EGFR play a pivotal role in disease progression. During progression of PC, COX-2, and 5-LOX are over-expressed, along with EGFR (Fig. 9). Studies showed that the activation of the EGFR pathway promotes transcription of the COX-2 gene [25, 26]. Likewise, the COX-2 signaling pathway activates EGFR phosphorylation [27] and EGFR transcription [28]. The EGFR and COX-2 pathways interact at several levels, and are involved in carcinogenesis, angiogenesis, and chemoresistance. The EGFR pathway plays a central role in the regulation of COX-2 expression. Activation of the Ras/Raf/mitogen-activated protein kinase and NF-κB transcription factor upregulates COX-2 gene transcription and the activation of the PI3K/Akt pathway stabilizes COX-2 mRNA [25, 26]. Similarly, overexpression of the COX-2 enzyme can potentially affect the EGFR signaling pathway. Prostaglandins (PG) transactivate the EGFR by induction of phosphorylation of the EGFR and extracellular signal-regulated kinase [27]. Further, COX-2 overexpression induces EGFR expression [28]. In addition to COX-2 inhibition, celecoxib is known to directly inhibit the EGFR pathway through inhibition of the PI3K/Akt and NF-κB pathways [29–31]. Although EGFR inhibitors are in use, their benefits are limited and the use of higher doses leads to undesirable side effects and toxicities. Clinically, targeting inflammation by selective COX-2 inhibitors leads to increased cardiovascular and GI toxicities. Therefore, targeting both EGFR and 5-LOX-COX- simultaneously may be an effective approach to modulate three pathways and their downstream signaling, which may result in an increased treatment response. Hence, the aim of this study was to determine whether simultaneous targeting of inflammatory pathways (5-LOX-COX) and EGFR at early stages would lead to blockade of progression of PanIN lesions to carcinoma.

We first used transcriptome analysis to establish that COX-2, 5-LOX, and EGFR concurrently overexpress in PDAC from GEM compared with pancreata from wild-type mice (Fig. 9A). We chose licofelone as anti-inflammatory agent due to its ability to simultaneously inhibit COX and 5-LOX, which limits undesirable cardiovascular and gastrointestinal side effects, and chose gefitinib as the EGFR inhibitor to study their individual and combined effects on PDAC development. Lower doses of these two agents were selected, based on our previous studies [5, 12]. In our long-term *in vivo* efficacy study, we demonstrated that licofelone and gefitinib can target COX-2, 5-LOX, EGFR, and cancer stem cell marker DclK1, and can potently inhibit PanIN progression to PDAC without any side effects.

Male and female mice receiving treatment with licofelone or gefitinib alone showed dramatic inhibition of PDAC incidence. A complete blockade of PDAC (100% inhibition) was observed with the combination treatment. Up to 70% of development of carcinoma *in situ* (PanIN 3 lesions) was blocked by the combination, compared with a 56% and 50% blockade by the individual drugs. These results clearly demonstrate that combination treatment effectively blocks progression of PanIN lesions to ductal adenocarcinoma. Further, about 70% of the pancreata from mice receiving the combination treatment were free from PanIN lesions.

We have previously shown *in vivo* chemopreventive effects of licofelone on colon and bladder cancers [32, 33]. There are limited reports on the evaluation of COX-2 inhibitors, and no reports on dual COX/5-LOX inhibitors used in combination with EGFR inhibitors against PC using a GEM model. For example, the use of selective COX-2 inhibitor nimesulide and NO-aspirin delayed the progression of PC precursor lesions in the Kras^G12D^ mouse model [2, 3]. In *in vitro* studies, the combination of celecoxib, SC-236, and NS-398 with gefitinib, trastuzumab, and cetuximab showed growth inhibition and potentiated the apoptotic effect in squamous cell cancer and breast and colon cancer cell lines [34–36].

The effects of the combination treatment on pancreatic tumor weights were strongly correlated with effects on PDAC incidence, carcinoma *in situ* development, and incidence of ductal adenocarcinoma. The pancreas weights from GEM receiving the combination treatment were comparable to those of wild-type mice. Previous investigations of our group and others revealed the anti-proliferative and apoptotic effects of licofelone and gefitinib on colon, bladder, and pancreatic cancers [5, 12, 32, 33]. Our results here clearly indicate that the licofelone+gefitinib combination effectively suppressed the growth and progression of PC in GEM. We propose that the synergism obtained due to simultaneous inhibition of 5-LOX-COX- and EGFR at early stages might be an underlying mechanism for the enhanced antitumor effects. *In vitro* studies of pancreatic cancer reported that a combination of erlotinib and celecoxib downregulated EGFR and COX-2 expression and activation [37]. However, further studies are warranted to establish cause and effect.

In the present study, licofelone+gefitinib suppressed DclK1 CSC marker and inhibited tumor progression, compared with individual drug treatments, suggesting that the combination may effectively act on eliminating CSCs. Though PGE2 is implicated in CSC survival and proliferation, recent evidence shows that 5-LOX is also a candidate for the management of stem cells [38]. We also observed that genetic ablation of COX-2 did not significantly reduce DclK1 in Kras GEM mice [5]. This may be partly attributed to the shift of arachidonic acid metabolism pathways towards 5-LOX upon selective inhibition of COX-2 [5]. Hence, the use of a dual 5-LOX-COX-inhibitor like licofelone is ideal to kill CSCs effectively. Drugs like metformin and licofelone have been shown to specifically target cancer stem cells, thereby showing greater effects in blocking pancreatic and breast tumor growth and prolonging remission [5, 17, 39, 40]. The concurrent inhibition of cyclooxygenase-2 and EGFR leads to greater anti-tumor activity in pancreatic cancer [37]. In addition to COX-2 inhibition, celecoxib is known to directly inhibit the EGFR pathway through inhibition of the PI3K/Akt and NF-κB pathways [37]. *In vitro* studies showed no potentiation in growth inhibition or apoptosis in the cell lines with low EGFR expression. However, significant downregulation of COX-2 and EGFR expression was observed in cells with high EGFR expression treated with the combination of erlotinib and celecoxib, compared with individual drugs [37]. Since high EGFR expression is seen in about 87% of patients with PC, and also reflected in the present study, the combination of licofelone and gefitinib reduced COX-2, 5-LOX, EGFR, DclK1, mPGES-1, mPGES-2, FLAP, and β-catenin, and increased p21 expression, compared with untreated controls and animals treated with either drug alone. Importantly, number of genes responsible tumor immune responses and desmoplastic reaction; Mst1r, TGFβr1, TGFβr2, VEGFa, CSF-1, SDF-2, CD44, IL-6ra, PD1, CD68, CD163, fibronection and MMPs (Tables 1 & 2) were significantly reduced. Whereas the mRNAs of genes that helps in tumor inhibition like Galr1, tumor suppressor Gnmt and Fn3k were significantly increased. Further, transcriptome data and PanIN lesion inhibition data obtained during the tumor progression stage corroborated with these results (Fig. 9B–9D). Although RNA seq data showed extensive changes with combination treatment, the gene expression pattern in the tissues did not return to normal after treatment. The reason for this could be due to presence of PanIN lesions even after combination treatment (Fig. 9D).

**Figure 10:**
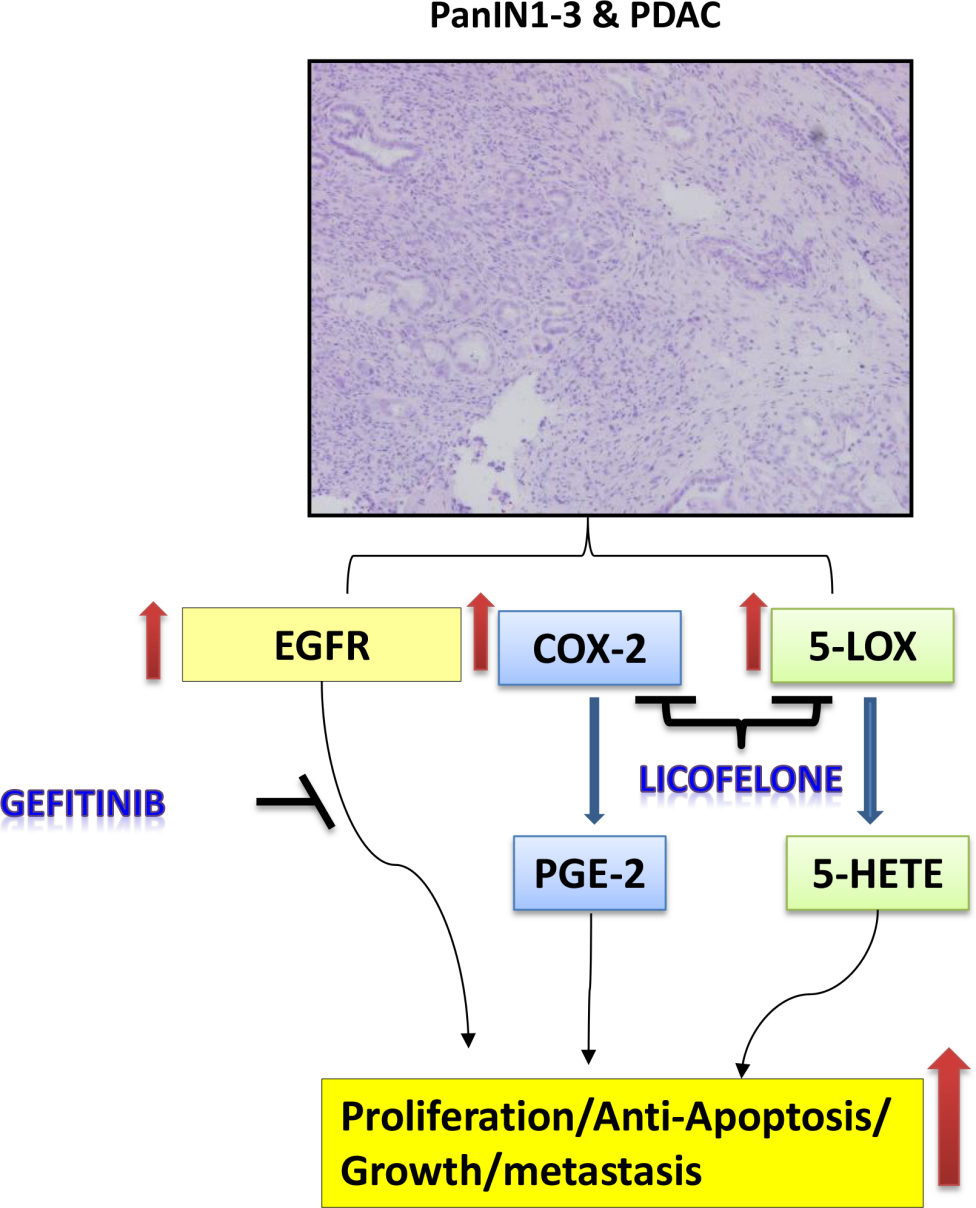
Schematic representation of effects of gefitinib and licofelone on PC progression Hematoxylin and Eosin staining showing PanINs and PDAC. Pathways showing the effects of gefitinib and licofelone on inhibition of cell proliferation and tumor progression.

Mechanistically, the effects of the gefitinib and licofelone combination can be attributed to the inhibition of expression of COX-2, 5-LOX, EGFR, and DclK1, as shown by the schematic diagram in Fig. 10. In addition, the combination significantly downregulated mPGES-1, mPGES-2, and FLAP, which in turn contribute to the release of prostaglandins (PG), leukotrienes, and thromboxane; and changes in the tumor immune responses and desmoplastic reaction. Further studies are warranted to evaluate the exact and in-depth mechanism of this combination treatment. In conclusion, licofelone can potentiate the pancreatic tumor growth inhibitory effects of gefitinib in GEM with pancreatic cancer. Our study reports for the first time the use of combination of a dual 5-LOX-COX- inhibitor, licofelone, with gefitinib in a genetically engineered Kras mouse model that recapitulates human disease progression in a step-wise manner. However, further studies using different GEMs must be conducted to translate this combination approach to the clinic. Therefore, targeting the EGFR and 5-LOX-COX- pathways seems to be a promising approach for the prevention and/or treatment of pancreatic cancer.

## MATERIALS AND METHODS

### Mouse model, diet, and handling

All animal research was performed under the auspices of animal protocols approved by the University of Oklahoma Health Sciences Center institutional animal care and use committee. Animals were housed in ventilated cages under standardized conditions (21°C, 60% humidity, 12-h light/12-dark cycle, 20 air changes/hour) in the University rodent barrier facility. Semi-purified modified AIN-76A diet ingredients were purchased from Bioserv, Inc., NJ. Generation of p48^Cre/+^-LSL-Kras^G12D/+^ mice expressing the activated Kras^G12D^ oncogene has been described previously [17, 18]. The dual COX/5-LOX inhibitor licofelone and EGFR inhibitor gefitinib were procured from the NCI-DCP chemoprevention drug repository. Mice were allowed *ad libitum* access to the respective diets (licofelone 250 ppm, gefitinib 100 ppm, or a combination) and to automated tap water purified by reverse osmosis.

### Breeding and genotyping analysis

LSL-Kras^G12D/+^ and p48^Cre/+^ mice were maintained in a C57BL/6 heterozygous genetic background. LSL-Kras^G12D/+^ and p48^cre/+^ mice were bred and offspring of activated p48^Cre/+^. LSL-Kras^G12D/+^ and C5BL/6 wild-type mice were generated at the required quantities. Briefly, genomic DNA was isolated from tail tissue samples using the mini-prep kit (Invitrogen, Carlsbad, CA). Polymerase chain reaction (PCR) was performed for K-ras and Cre genes using the following conditions: denaturation at 95°C for 5 min, followed by 35 cycles at 95°C for 1 min, 60°C for 1 min, and 72°C for 1 min. Oligonucleotide primer sequences used were as follows: K-ras 5′-CCTTTACAAGCGCACGCAGAG-3′ sense, 5′-AGCTAGCCACCATGGCTTGAGTAAGTCTGCA-3′ anti-sense; and p48Cre 5′-ACCGTCAGTACGTGAGAT ATCTT-3′ sense and 5-ACCTGAAGATGTTCGCGATTA TCT-3′ antisense. PCR products were separated on a 2% agarose gel. Successful recombination yields were 550 and 350-bp products for Kras and Cre genes respectively (17). The genotype of each pup was confirmed by tail DNA extraction and PCR.

### Preclinical assay: efficacy of licofelone

Genotyped male and female p48^Cre/+^-LSL-Kras^G12D/+^ transgenic mice were used in the efficacy study. Five-week-old mice were selected and randomized so that average body weights in each group were equal. Mice were fed AIN-76A diet for one week. At 6 weeks of age, p48^Cre/+^-LSL-Kras^G12D/+^ mice were fed AIN-76A experimental diets containing 0 ppm drug (*n* = 34/group + *n* = 12 C57BL/6 wild-type mice), 250 ppm (*n* = 28/group) licofelone, 100 ppm (*n* = 23/group) gefitinib, or a combination of licofelone+gefitinib (250 ppm + 100 ppm, *n* = 24/group) until termination of the study. After 38 weeks (~10 months) on experimental diets, all mice were euthanized by CO_2_ asphyxiation and necropsied. Pancreata were collected from all groups, weighed, and snap-frozen in liquid nitrogen for further analysis. Pancreata (head to tail) required for histopathologic and IHC evaluations to identify PanIN lesions and PDAC for evaluation of various molecular markers were fixed in 10% neutral-buffered formalin, as previously described.

### Tissue processing and histological analysis of PanIN lesions and PDAC

After euthanizing the mice, pancreata and other key organs, including liver, spleen, kidney, and lung, were collected and weighed. Tissues were fixed in 10% formalin for 24 h and routinely processed and embedded in paraffin. Formalin-fixed 4-μm tissue sections of each pancreas were stained with Hematoxylin & Eosin (H&E) and were histologically evaluated by a pathologist blinded to the experimental groups. PanIN lesions and carcinoma were classified according to histopathologic criteria as recommended elsewhere [4, 12–18]. To quantify the progression of PanIN lesions, the total number of ductal lesions and their grades were determined [12–18]. Similarly, pancreatic carcinoma and normal appearing pancreatic tissue were evaluated in all animals.

### Immunohistochemistry (IHC) and immunofluorescence (IHF)

Fixed 5-μm sections were incubated with primary antibodies in a hybridization chamber for 1 h at room temperature or overnight at 4°C. The primary antibodies were proliferating cell nuclear antigen (PCNA), COX-2, 5-LOX, and pEGFR procured from Santa Cruz/Abcam/Cell Signaling. Following incubation with primary antibody, sections were incubated for 1 h with anti-mouse/anti-rabbit/anti-goat secondary antibody, then were visualized with diaminobenzidine (DAB) and were counterstained with hematoxylin for IHC or with DAPI for IHF. Slides were observed under an Olympus microscope 1X701. Digital computer images were recorded with an Olympus DP70 camera.

### Apoptosis assay

Paraffin sections (*n* = 6 mice/group) of 5-μm thickness that had been mounted on slides were rehydrated and stained using the Fragment End Labeling (FragEL) DNA Fragmentation Detection Kit with the TUNEL method, following the manufacturer's instructions (Millipore, Billerica, MA). This kit allows the recognition of apoptotic nuclei in paraffin-embedded tissue sections fixed on slides by FragEL of DNA. The terminal deoxynucleotidyl transferase binds to exposed ends of DNA fragments generated in response to apoptotic signals, and catalyzes the template-dependent addition of biotin-labeled and biotin-unlabeled deoxynucleotides. Biotinylated nucleotides are detected using streptavidin-HRP conjugate. Diaminobenzidine reacts with the labeled sample to generate an insoluble colored product at the site of DNA fragmentation. Counter-staining with methyl green aids in the morphologic evaluation and characterization of normal and apoptotic cells. Stained apoptotic epithelial cells (a minimum of 10 microscopic fields per section) were counted manually in a single-blind fashion.

### Western blot analysis

Proteins (60 ug) in lysates from pancreata from control and drug-treated mice were separated by SDS-PAGE and were transferred to a nitrocellulose membrane. Membranes were blocked with 5% nonfat milk (Biorad) in Tris-buffered saline (TBS), and were then incubated with antibodies for PCNA, COX-2, 5-LOX, Dclk1, p21, β-catenin, and actin overnight at 4°C. Subsequently, membranes were washed and incubated with HRP-secondary antibody for 1 h. Protein was detected on BioMax MR film (Kodak) using chemiluminescence (Super Signal, Pierce Biotechnology). Equal protein loading was confirmed by detection of actin. ImageJ was used to perform image analysis.

### Next generation sequencing (NGS)/RNA-seq and data analyses

Pancreatic tumor tissues were collected from at least three untreated control mice and three combination-drug-treated mice, after 25 weeks of drug treatment. Total RNA was extracted with Trizol reagent (Life Technologies, San Francisco, CA) and subjected to cDNA library construction, followed by NGS, per the NGS protocol, with an Illumina sequencer in the OUHSC core facility (Microgen). The sequence data was deposited to the Geospiza/Perkin Elmer (Seattle, WA) company server and analyzed with GeneSifter bioinformatics software. The same data set was also analyzed with Strand bioinformatics software (Strand-NGS, San Francisco, CA).

### Statistical analysis

The data are presented as means ± *SE*. Differences in body weights were analyzed by *ANOVA*. Statistical differences between control and treated groups were evaluated using Fisher's exact test for PDAC incidence. Unpaired *t*-test with Welch's correction was used for PanIN and PDAC lesions. Differences between groups are considered significant at *p* < 0.05.

## SUPPLEMENTARY FIGURE


