# Supplementary material for: Simultaneous targeting of 5-LOX-COX and EGFR blocks progression of pancreatic ductal adenocarcinoma
Source: Oncotarget. 2015 Sep 28;6(32):33290–305. doi: 10.18632/oncotarget.5396 (PMC4741766; doi:10.18632/oncotarget.5396)
Supplement: Supplementary file 1 [file oncotarget-06-33290-s001.pdf]

SUPPLEMENTARY FIGURE

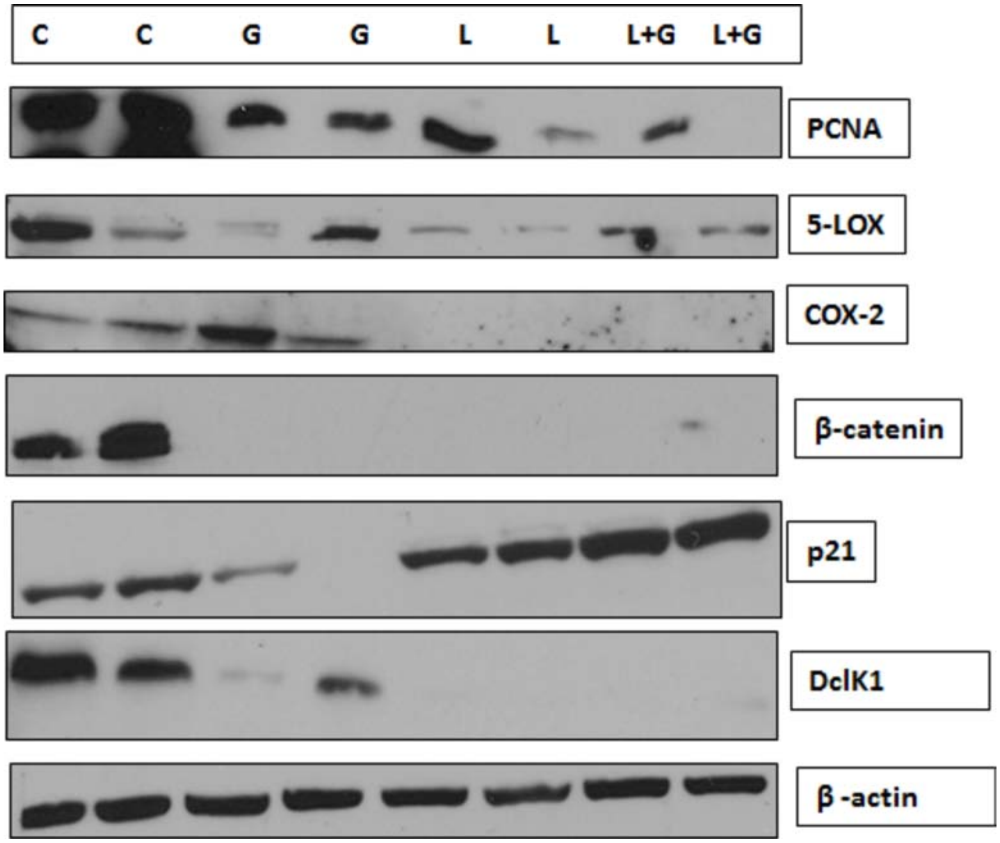

Supplementary Figure S1: Western immunoblotting results showing the effect of L, G, and L+G on expression of PCNA, 5-LOX, COX-2, β-catenin, DclK1, and p21, with β-actin as loading control.
